# Circadian Clocks and Pregnancy

**DOI:** 10.3389/fendo.2014.00123

**Published:** 2014-07-24

**Authors:** James Olcese

**Affiliations:** ^1^Department of Biomedical Sciences, Florida State University College of Medicine, Tallahassee, FL, USA

**Keywords:** circadian, circadian clocks, pregnancy, reproductive systems, cellular circadian clock

The recognition that 24-h rhythmic processes (“circadian”) underlie many endocrine functions has added a fascinating new temporal dimension to our appreciation of their complexity. Research from various laboratories has revealed circadian gene expression in multiple tissues in the reproductive system of non-pregnant and pregnant mammals. However, many questions regarding role of circadian clocks in the female reproductive system remain unresolved. This Research Topic on *Circadian Clocks and Pregnancy* brings together a broad and innovative group of researchers to discuss, evaluate, and summarize the key aspects of circadian function as it relates to all aspects of pregnancy from ovulation and implantation to fetal development and parturition.

In the initial review, Ditisheim et al. (1) address preeclampsia, a pregnancy-related disease that involves an imbalance of angiogenic factors, which leads to abnormal placentation, marked systemic inflammatory responses, and hypertension. One particularly relevant feature of this disease is the loss of the nocturnal decrease in arterial blood pressure. The authors assess the question of whether disruption of circadian function may represent an occupational risk factor for preeclampsia and make the case that while this condition should be considered a major public health concern, the evidence for shift work increasing the risk of preeclampsia is not yet persuasive enough to draw any solid conclusions. Using a DNA microarray approach, Tasaki et al. (2) analyzed gene expression in uterine endometrial stromal cells during the implantation stage in the rat. In particular, they focused on the evaluation of genes compromising the circadian clockwork as well as downstream clock-controlled genes, since mutations in the former are known to associate with implantation failure and other reproductive defects. Among their findings was evidence for the clock gene Bmal1 controlling inhibin-β, IGF-1, and other endometrial genes. They also developed the hypothesis that alterations in the circadian system during decidualization are important for regulating the expression of genes vital for the formation of the placenta. In his review, Sellix (3) highlights our current understanding of circadian clock functions in the female reproductive system as it relates to the timing of gonadotropin secretion at ovulation and the timing of parturition. He proposes that the neuroendocrine-reproductive axis should be considered a partnership of synchronized and cooperative oscillators whose disruption can help to explain some forms of fertility disorders. In their contribution, Gamble et al. (4) examine the importance of circadian clocks for successful pregnancy. It is well known that shift work increases the risk of miscarriages, preterm birth, low birth weights, etc. These authors identify key gaps in our knowledge of circadian clock function in reproductive physiology and offer useful strategies for better understanding how shift work-induced circadian misalignment leads to reproductive dysfunctions. The complex relationship between cholesterol metabolism, estrogens, and female reproductive cycles is reviewed by Urlep and Rozman (5). For example, various genes in the cholesterol and steroidogenic pathway are rhythmic and are regulated by clock genes. Conversely, the central circadian clock in the suprachiasmatic nuclei (SCN) expresses the estrogen receptor, ERβ, which is also under clock gene control. Thus, perturbations in clock gene expression can have profound effects at multiple levels of the neuroendocrine-reproductive axis. Watanabe et al. (6) address the growing recognition that circadian rhythms during pregnancy have important consequences on fetal development. In their interesting clinical trials, they have determined that preterm babies (week 31 of pregnancy) already have pupillary reflexes and that exposure of these infants to a regular light/dark cycle (as opposed to the more common constant light environment of neonatal units) promotes more daytime activity and body growth. In other words, the direct use of natural circadian cycles of light and dark would appear to offer the preterm infant a surrogate rhythmic environment more conducive to optimal postnatal development, underscoring the importance of the circadian system during pregnancy. While it has long been recognized that female reproductive cycles are under the control of the central circadian clock in the SCN, only in the last few years have the specific details of this hypothalamic regulation been clarified. Miller and Takahashi (7) provide a lucid overview of the contributions of circadian vasopressin signals to the kisspeptin neurons that regulate hypothalamic GnRH secretion as well as the VIP regulation of pituitary prolactin rhythms. They also make a compelling case for the effects of clock gene mutations on pregnancy having important similarities to the impact of senescence on reproductive abnormalities.

As documented by this Research Topic, the study of cellular circadian clocks on reproductive systems is still fertile territory for endocrinologists, cell biologists, and clinicians alike. It has great potential for providing testable hypotheses and unified explanations for disparate processes, such as blood pressure and gene expression rhythms, effects of shift work and artificial lighting on fetal development, hormonal rhythms and fertility, etc. An exciting era awaits!

## Conflict of Interest Statement

The author declares that the research was conducted in the absence of any commercial or financial relationships that could be construed as a potential conflict of interest.

## References

[1] Ditisheim AJ Dibner C Philippe J Pechère-Bertschi A Biological rhythms and preeclampsia. Front Endocrinol (2013) 4:47 10.3389/fendo.2013.00047 23579266 PMC3619120

[2] Tasaki H Zhao L Isayama K Chen H Yamauchi N Shigeyoshi Y Profiling of circadian genes expressed in the uterus endometrial stromal cells of pregnant rats as revealed by DNA microarray coupled with RNA interference. Front Endocrinol (2013) 4:82 10.3389/fendo.2013.00082 23847593 PMC3703733

[3] Sellix MT Clocks underneath: the role of peripheral clocks in the timing of female reproductive physiology. Front Endocrinol (2013) 4:91 10.3389/fendo.2013.00091 23888155 PMC3719037

[4] Gamble KL Resuehr D Johnson C Shift work and circadian dysregulation of reproduction. Front Endocrinol (2013) 4:92 10.3389/fendo.2013.00092 23966978 PMC3736045

[5] Urlep Z Rozman D The interplay between circadian system, cholesterol synthesis, and steroidogenesis affects various aspects of female reproduction. Front Endocrinol (2013) 4:111 10.3389/fendo.2013.00111 24065951 PMC3778439

[6] Watanabe S Akiyama S Hanita T Li H Nakagawa M Kaneshi Y Designing artificial environments for preterm infants based on circadian studies on pregnant uterus. Front Endocrinol (2013) 4:113 10.3389/fendo.2013.00113 24027556 PMC3761559

[7] Miller BH Takahashi JS Central circadian control of female reproductive function. Front Endocrinol. (2014) 4:195 10.3389/fendo.2013.00195 PMC3898595 24478756

